# Syndecan-4 Functionalization Reduces the Thrombogenicity of Engineered Vascular Biomaterials

**DOI:** 10.1007/s10439-023-03199-w

**Published:** 2023-04-18

**Authors:** Yidi Wu, William D. Wagner

**Affiliations:** 1 Department of Plastic & Reconstructive Surgery, Medical Center Boulevard, Wake Forest University School of Medicine, Winston-Salem, NC 27157 USA; 2 Virginia Tech - Wake Forest University School of Biomedical Engineering and Sciences, Winston-Salem, NC USA; 3 Wake Forest Institute for Regenerative Medicine, Wake Forest School of Medicine, 391 Technology Way, Winston-Salem, NC USA

**Keywords:** Vascular biomaterial, Syndecan-4, Thrombogenicity

## Abstract

Blood–biomaterial compatibility is essential for tissue repair especially for endovascular biomaterials where small-diameter vessel patency and endothelium formation is crucial. To address this issue, a composite biomaterial termed PFC fabricated from poly (glycerol sebacate), silk fibroin, and collagen was used to determine if functionalization with syndecan-4 (SYN4) would reduce thrombogenesis through the action of heparan sulfate. The material termed, PFC_SYN4, has structure and composition similar to native arterial tissue and has been reported to facilitate the binding and differentiation of endothelial colony-forming cells (ECFCs). In this study, the hemocompatibility of PFC_SYN4 was evaluated and compared with non-functionalized PFC, electrospun collagen, ePTFE, and bovine pericardial patch (BPV). Ultrastructurally, platelets were less activated when cultured on PFC and PFC_SYN4 compared to collagen where extensive platelet degranulation was observed. Quantitatively, 31% and 44% fewer platelets adhered to PFC_SYN4 compared to non-functionalized PFC and collagen, respectively. Functionalization of PFC resulted in reduced levels of complement activation compared to PFC, collagen, and BPV. Whole blood clotting times indicated that PFC_SYN4 was less thrombogenic compared with PFC, collagen, and BPV. These results suggest that syndecan-4 functionalization of blood-contacting biomaterials provides a novel solution for generating a reduced thrombogenic surface.

## Introduction

Cardiovascular diseases (CVDs) remain the leading cause of death in the United States, resulting in the death of 1 in 4 people [1, 2]. Atherosclerotic coronary artery disease (CAD) is the most common type of CVD, affecting over 18 million adults [3]. The gold standard of treatment for CAD is Coronary Artery Bypass Grafting (CABG), which is widely performed to bypass the occluded region and improve blood flow.

There is an unmet clinical need for small-diameter (< 6 mm) vascular grafts in CABG procedures for CAD patients. Currently, autologous saphenous vein grafts (SVGs) are the most commonly used grafts for small-diameter CABG surgery [4]. However, suitable vein grafts are not always available, and the harvesting procedure is invasive. More importantly, the failure of vein grafts in the long term due to compliance mismatch remains an unsolved problem. SVG occlusion rates are estimated to be ~ 50% within 10 years [5, 6]. The internal mammary artery (IMA) is the current gold standard conduit for CABG. The 10-year patency rate is around 90% [4]. However, donor site wound complications remain a major concern and suitable vessels are not always available, especially for elderly patients or patients with chronic diseases [7, 8]. While clinically available expanded polytetrafluoroethylene (ePTFE, Teflon®) and polyethylene terephthalate (PET, Dacron®) grafts have been successfully used in large diameter arterial procedures, they have poor performance in small-diameter CABG surgery [9]. Unlike native vessels, these grafts are rigid and lack elasticity. The mismatch in viscoelastic properties results in turbulent flow post-implantation, which ultimately causes serious complications such as thrombosis and stenosis. For pediatric patients, these non-degradable grafts lack growth and remodeling potential, so repetitive surgical interventions are often needed after implantation. The surface properties of these non-degradable materials are not suitable for cellular adhesion and infiltration and the grafts become thrombotic due to a lack of functional endothelium. The best treatment option for each patient usually needs to be customized, which drives the development of new technologies [10].

Several types of decellularized grafts are commercially available including Artegraft®, Procol®, and CryoVein® [11]. However, the host immune response due to remaining graft cellular components as well as insufficient mechanical strength limits the clinical use of these grafts [12]. To address the clinical need and prevent postoperative graft failure, biodegradable polymers have been used to fabricate vascular grafts as alternatives to currently used synthetic grafts. Poly(glycerol sebacate) (PGS), polycaprolactone (PCL), and poly(l-lactic acid) (PLLA) are often used in composite biomaterials to create vascular tissue extracellular matrix mimetics [13–15]. Silkothane® is a commercialized electrospun vascular graft composed of silk fibroin and polyurethane. The mechanical properties of Silkothane® grafts can be tuned by varying weight ratios of silk fibroin and polyurethane resulting in a Young’s modulus ranging between 4 and 23 MPa [16]. It has been reported the grafts remained patent as evaluated by arteriovenous shunt in a sheep model for up to 90 days [17]. We have previously reported a viscoelastic vascular composite material composed of PGS, silk fibroin, and type I collagen, termed PFC [18]. The biomaterial is mechanically durable with structural and compositional similarities to native vascular tissue. It has been shown to be minimally thrombogenic, support endothelial cell growth, and withstand physiological pressure [18].

In order to promote long-term patency, surface functionalization is usually necessary to reduce the thrombogenicity of prosthetic vascular grafts. Hemocompatibility is one of the most important surface properties for blood-contacting biomaterials and determines the in vivo performance and long-term success of the engineered biomaterial after implantation [19]. Many surface modification strategies have been developed to promote biomaterial surface hemocompatibility and improve the long-term patency of the biomaterials after surgical implantation [20]. Heparin has been widely used as an active anticoagulant surface treatment to inhibit intrinsic thrombogenicity and improve the hemocompatibility [21]. However, heparin-modified surfaces are not the ideal solution for long-term use with major problems that include biocompatibility and burst release of heparin [22]. Nonspecific binding of plasma protein often occurs due to highly sulfated linear polysaccharides in heparin [23]. Undesirable side effects such as increased risk of heparin-induced thrombocytopenia (HIT) remain a concern [24].

Syndecan-4 is a cell surface proteoglycan with three heparan sulfate (HS) glycosaminoglycans having binding sites for a variety of cellular growth and signaling molecules. Syndecan-4 is naturally present in the endothelial glycocalyx, which coordinates the interaction with anticoagulation molecules and maintains the non-thrombogenic property of the endothelium [25]. The molecular domain and spatial properties of syndecan-4 provide more variations in oligosaccharide sequences in HS compared with heparin [26]. The structural diversity of HS facilitates the specific interactions between syndecan-4 and over 400 cellular signaling molecules, including anticoagulant molecules, growth factors, and cell adhesion molecules [27]. HS chains facilitate the binding of ECM proteins, including fibronectin and plasma proteins such as antithrombin, critical for inhibiting several enzymes of the coagulation cascade [28–30]. Additionally, the specific binding sequences for various growth factors have been identified, including fibroblast growth factors (FGF), vascular endothelial growth factors (VEGF), and platelet-derived growth factors (PDGF) [31, 32]. In a previous study, we demonstrated syndecan-4 significantly enhanced the binding of growth factors to PFC and resulted in an increased capture of endothelial colony-forming cells (ECFCs) [33]. In a further study, we demonstrated syndecan-4 functionalization of PFC promoted spreading and differentiation of ECFCs [34].

The use of syndecan-4 can increase the binding specificity and facilitate the interaction with various cell signaling molecules compared with heparin. This may create a unique solution and meet the need for in situ supply or delivery of these molecules, resulting in stabilization and protection from proteolytic degradation [35, 36]. Additionally, syndecan-4 may not cause serious adverse effects such as HIT as seen in heparin-functionalized biomaterials. Thus, we hypothesize that the use of syndecan-4 for surface modification may provide a novel solution for creating hemocompatible biomaterials. The use of anticoagulant therapy and side effects may also be reduced by creating a more hemocompatible biomaterial surface [37]. We previously reported a preliminary study on platelet interaction with PFC and found platelets were less activated on PFC compared with collagen [18]. In the current study, we conducted a comprehensive evaluation of the hemocompatibility of syndecan-4 functionalized PFC compared with PFC, collagen, commercially available ePTFE, and bovine pericardial patch. The purpose of the current study was to functionalize a compliance-matched vascular graft material, PFC, with heparan sulfate containing syndecan-4 (PFC_SYN4) and demonstrate improvement in the non-thrombogenic property. The results of this study provide a potentially novel solution in the creation of a hemocompatible vascular biomaterial to manage adverse thrombotic events.

## Materials and Methods

### Materials

Poly(glycerol sebacate) (PGS) prepolymer was synthesized from sebacic acid (Sigma-Aldrich) and glycerol (Fisher Scientific) using a published protocol [38]. Type I collagen was purchased commercially (Elastin Products Company, Inc). Silk fibroin was extracted from raw silk (Haian Silk Company, Nantong, China) according to published methods [39]. Recombinant human syndecan-4 was obtained commercially (R&D Systems). The human complement C3 ELISA kit and the human complement C3a des Arg ELISA kit were purchased from Abcam. The lactate dehydrogenase (LDH) activity assay kit was purchased from Sigma-Aldrich. ePTFE vascular grafts were obtained from GORE-TEX® (Newark, Delaware). Bovine pericardial patch for valve replacement (BPV) was obtained from Edwards Lifesciences Corp. (Irvine, CA).

### Scaffold Fabrication and Functionalization

**Fig. 1:**
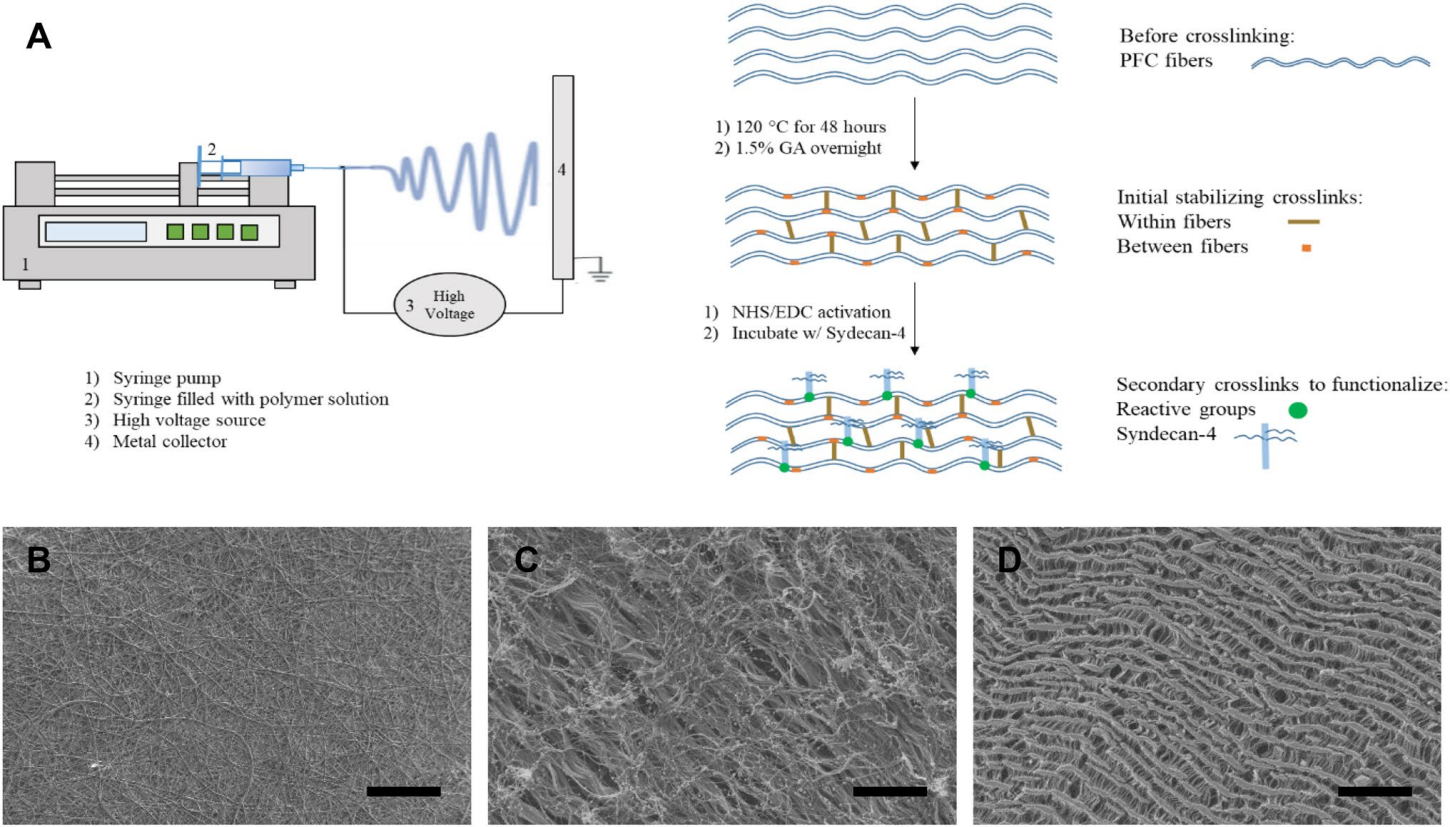
**A** An illustrative diagram of electrospinning set up and material fabrication and functionalization of PFC. **B**–**D** Representative scanning electron microscopy images of PFC (**B**) compared with commercially available endovascular materials, bovine pericardial patch (**C**) and ePTFE (**D**) at 1000×. (Scale bar = 100 µm)

A composite of poly(glycerol sebacate) (PGS), silk fibroin, and type I collagen was dissolved in 1,1,1,3,3,3-hexafluoro-2-propanol (HFIP) (10% w/v ratio) (Sigma-Aldrich) at a mass ratio of 4.5:4.5:1. The polymer solution was stirred overnight and then loaded into a 5-ml syringe with an 18-gauge blunt tip needle. Then a 25 kv voltage was applied to the needle tip and the solution was electrospun onto a metal collector at 2 ml/h using a syringe pump (Baxter, Model AS50). After electrospinning, the material was removed from the collector and incubated in an oven at 120 °C for 48 h to polymerize the PGS component. The material was treated with 1.5% glutaraldehyde (Sigma-Aldrich) vapor overnight and washed with 0.02 M glycine (Sigma-Aldrich) twice for 30 min to inactivate unreacted aldehydes as published previously [18]. The material, termed PFC, was functionalized with syndecan-4 using the two-step NHS/EDC method and termed PFC_SYN4 [40]. Briefly, PFC was incubated with 30 mM EDC (Thermo Scientific) and 6 mM NHS (Sigma-Aldrich) in MES buffer (0.1 M MES (4-Morpholineethanesulfonic acid) (Sigma-Aldrich), 0.5 M NaCl (Fisher Scientific), pH 6.0) for 30 min at room temperature. After incubation, PFC was rinsed in MES buffer and incubated with syndecan-4 (0.8 µg/cm^2^ PFC, as determined by the saturation curve based on Langmuir adsorption isotherm) in PBS for 2 h at 37 °C with agitation. The scaffolds were removed and rinsed with 0.1 M Na_2_HPO_4_ (Fisher Scientific) and PBS. The material fabrication and functionalization procedures of PFC are illustrated in Fig. 1A. The microarchitecture of PFC fibers (Fig. 1B) was compared with two commercially available endovascular materials, including bovine pericardial patch for valve replacement (Fig. 1C) and ePTFE for bypass surgery (Fig. 1D). The presence of syndecan-4 on PFC was confirmed with ELISA. All materials were prepared as electrospun mats of PFC, PFC_SYN4, or collagen for use in experiments.

### Preparation of Blood Samples

Platelet-rich plasma (PRP) and platelet-poor plasma (PPP) were prepared by standard centrifugation protocols using citrated fresh pig blood obtained from control pigs in another study. PRP was obtained by centrifuging whole blood at 200×*g* for 20 min. PPP was obtained from the upper pale yellow liquid layer by centrifuging whole blood at 2000×*g* for 10 min.

### Platelet–Material Interaction

Platelet activation on PFC, PFC_SYN4 and collagen eletrospun mats (*N* = 3) was evaluated by quantifying the number of adhered platelets and morphology. Materials were cut and fitted into a 96-well tissue culture plate for the following experiment. 200 µl PRP was added to the materials and incubated at 37 °C for 30 min under static conditions. After incubation with PRP, the suspension was aspirated and each sample was rinsed with PBS for three times and fixed with 2.5% glutaraldehyde in PBS. Then the samples were dehydrated using a series of ethanol solutions (50%, 60%, 75%, 80%, 90%, 95%, 100%) and further dried using hexamethyldisilazane (HMDS) (Thermo Scientific), then coated with a thin layer of gold. The morphology of adhered platelets in each sample was evaluated using scanning electron microscopy (Zeiss Gemini SEM 300). The diameter of adhered platelets and the number of adhered platelets were quantified from SEM images (*N* = 24) using ImageJ.

In an additional experiment, PRP was added to PFC, PFC_SYN4, BPV, ePTFE, and collagen (*N* = 3) and incubated for 1 h at 37 °C. BPV was used as a positive control and ePTFE was used as a negative control for the experiment. The number of adhered platelets on the materials was measured using the Lactate Dehydrogenase Activity Assay Kit (Sigma-Aldrich) according to the manufacturer’s protocol. After incubation with PRP for 1 h, the suspension was aspirated from each well, and the non-adherent platelets were rinsed away by filling and aspirating the wells three times with PBS. The adherent platelets were lysed with 1% Triton X-100 and LDH activity was measured as a quantitative evaluation of adhered platelets by recording the absorbance at 340 nm.

### Factor XII Activation

Materials were cut and fitted into a 96-well tissue culture plate for the following experiment. 200 µl of PPP was added to PFC, PFC_SYN4, BPV, ePTFE, and collagen (*N* = 3) and incubated for 30 min at 37 °C. After incubation with PPP, factor XII activation on the material surface was measured by adding the material to 200 µl substrate solution (0.3 mM chromogenic substrate, S-2302, solution in 50 mM HEPES, 120 mM NaCl) and incubated for 30 min. Factor XIIa activity was measured by the change in absorbance due to the amidolytic hydrolysis of the chromogenic substrate. The reaction was stopped with 20% acetic acid (Sigma-Aldrich) and the absorbance was measured at 405 nm.

### Complement Activation

Materials were cut and fitted into a 96-well tissue culture plate for the following experiment. 200 µl plasma was incubated with PFC, PFC_SYN4, BPV, ePTFE, and collagen (*N* = 3) at 37 °C for 30 min. Then the amount of Complement C3 and C3a in the plasma was quantified using the C3 and Human Complement C3a des Arg ELISA Kit (Abcam) with proper dilution of the samples according to the manufacturer’s protocol.

### Whole Blood Clotting

The thrombogenicity of the materials was evaluated using whole blood clotting time. PCL, PFC, PFC_SYN4, BPV, ePTFE, and collagen samples (*N* = 3) were cut and placed into 24-well tissue culture plates for the following experiment. The materials were hydrated following an incubation in PBS for 30 min. Recalcified pig whole blood (0.1 M CaCl_2_) was carefully added to the center of the hydrated materials at room temperature. 400 µl ddH_2_O was added to each sample at the end of each incubation time and incubated for 5 min to lyse the free red blood cells that were not trapped in a thrombus, thus releasing hemoglobin into the water for measurement. The supernatant was transferred to a 96-well plate and absorbance measured at 540 nm. The absorbance measurement of each sample was converted to percentage clotting by normalizing the absorbance with completely lysed whole blood samples (0% clotting) and ddH_2_O (100% clotting).

### Statistical Analysis

Group means adopted from pre-experiment or estimated from published studies and a power of 0.8 were used to calculate sample sizes. Statistical analyses were performed using GraphPad Prism software (version 9.2.0) using either a Student’s *t*-test or a one-way analysis of variance (one-way ANOVA) depending on the study design. Results were presented as mean ± standard error of the mean for each experiment group. If the results were significant (*P* < 0.05), a Tukey’s post hoc test was conducted to separate significant differences in means.

## Results

### Platelet Interaction

**Fig. 2:**
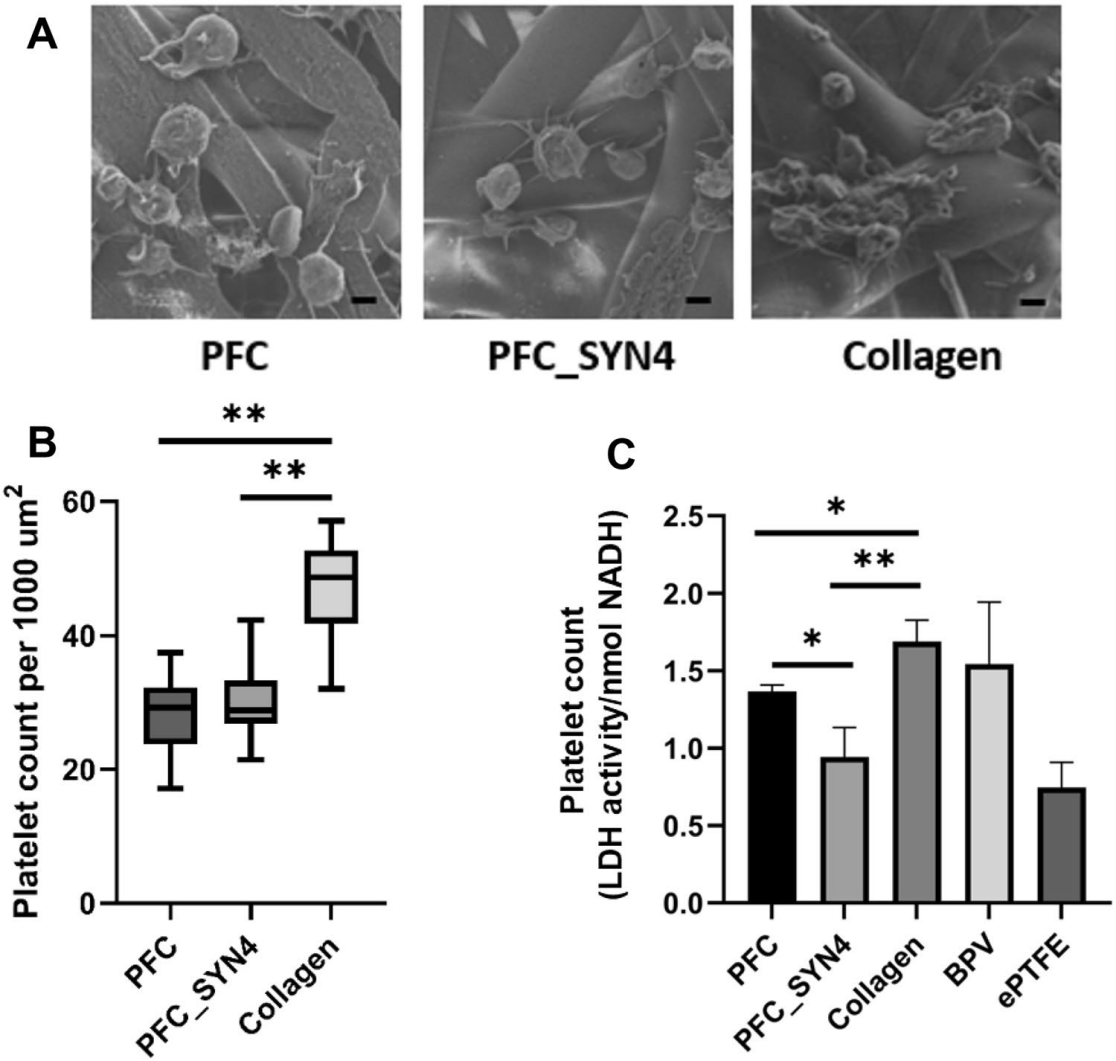
**A** SEM of platelet morphology on electrospun mats at 50,000× (scale bar = 1 μm). PRP was incubated with the materials for 30 min. **B**, **C** Quantification of platelets adhered on materials. **B** After 30-min incubation at 37 °C, the number of adhered platelets was quantified from SEM images. **C** After 1-h incubation at 37 °C, platelet adhesion was quantified using a lactate dehydrogenase (LDH) assay. (*P < 0.05, **P < 0.01)

The exposure of biomaterial to the blood can trigger the extrinsic coagulation pathway through the activation of platelets, which may lead to thrombotic events. An initial study was performed to see whether syndecan-4 functionalization was effective in reducing platelets interaction with PFC. After 30-min incubation, the platelets appeared to be less activated on PFC and PFC_SYN4 compared with collagen as shown by SEM assessment in Fig. 2A. The morphology of the platelets on PFC and PFC_SYN4 was rounded with defined borders. No morphological signs of activation were observed. In contrast, many platelets adhered to electrospun collagen with signs of platelet activation such as spreading, degranulation, and aggregate formation. The average diameter of adhered platelets for PFC_SYN4 (1.68 ± 0.1 µm) was significantly smaller (*P* < 0.01) compared with collagen (2.00 ± 0.06 µm). There was no statistical difference between PFC_SYN4 (1.68 ± 0.1 µm) and PFC (1.85 ± 0.08 µm).

**Fig. 3:**
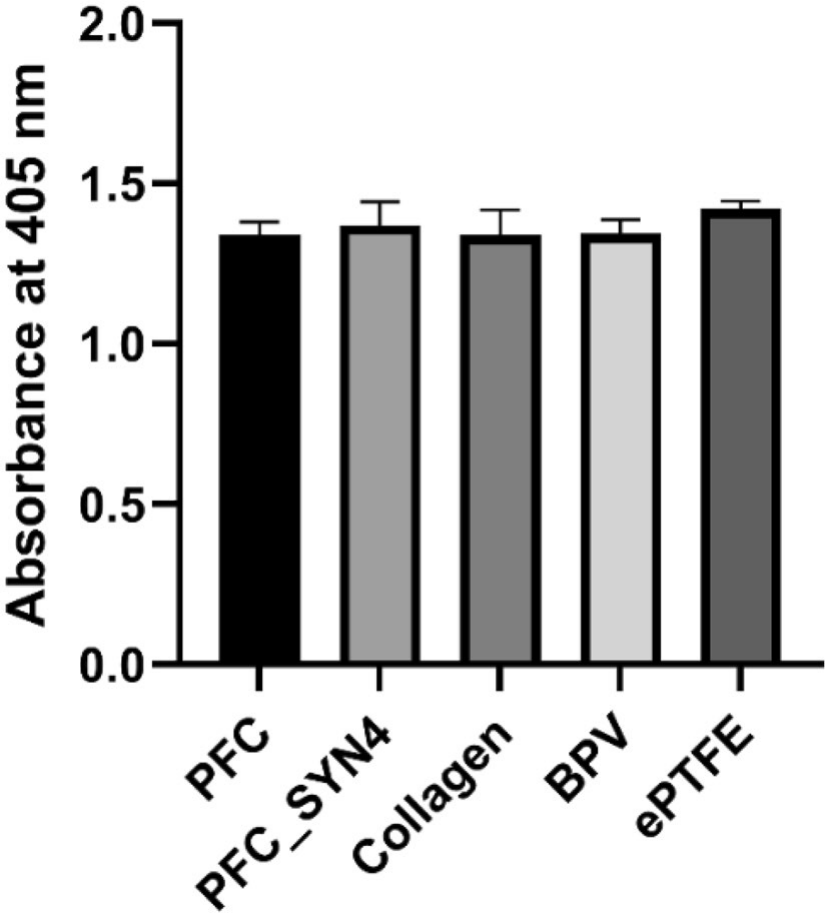
Factor XII activation analysis on the materials using a chromogenic substrate (S-2302). There was no statistical difference between the groups

The number of adhered platelets quantified from SEM images also indicated less platelet interaction with both types of PFC. There was a significant difference (*P* < 0.01) in the number of adhered platelets between collagen and both types of PFC mats as shown in Fig. 3A (*N* = 24). Collagen (47.7 ± 1.2) had significantly more adhered platelets per 1000 µm^2^ than PFC (28.2 ± 1.2) and PFC_SYN4 (29.9 ± 0.9).

To further examine and compare the level of platelet interaction on PFC and PFC_SYN4, lactate dehydrogenase activity was quantified and compared with ePTFE and BPV as shown in Fig. 2B. In this study, LDH level was a relative quantitative measurement of the amount of adhered platelets [41].

When the platelets were incubated with the materials for a longer period of time (1-h incubation), less platelets were found to adhere to PFC_SYN4 compared to PFC (*P* < 0.05) and compared to collagen (*P* < 0.01). LDH activity on PFC_SYN4 (0.94 ± 0.19 nmol) was significantly lower than PFC (1.37 ± 0.04 nmol) and collagen (1.69 ± 0.14 nmol). After incubation with PRP, collagen and BPV (1.54 ± 0.4 nmol) had the greatest number of adhered platelets. The least amount of adhered platelets was observed with ePTFE (0.75 ± 0.16 nmol) as expected. PFC functionalized with syndecan-4 had platelet adhesion similar to ePTFE.

### Factor XII Analysis

The intrinsic blood coagulation pathway initiated by contact activation is triggered by the interaction of plasma protein with an artificial biomaterial surface. The contact activation leads to the conversion of factor XII to an active enzyme factor XIIa, which further interacts with a series of clotting factors in the coagulation cascade. The amount of factor XIIa is an indicator of intrinsic coagulation pathway activation. In order to further characterize the activation of the intrinsic pathway, direct quantification of the catalytic activity of factor XIIa (the activated form of factor XII) was performed using a chromogenic substrate (S-2302). The chromogenic substrate can be broken down by factor XIIa and results in a change in absorbance. No significant difference between the groups was observed for factor XII activation as shown in Fig. 3, which indicated syndecan-4 functionalization of PFC did not activate the intrinsic blood coauglation pathway.

### Complement Analysis

**Fig. 4:**
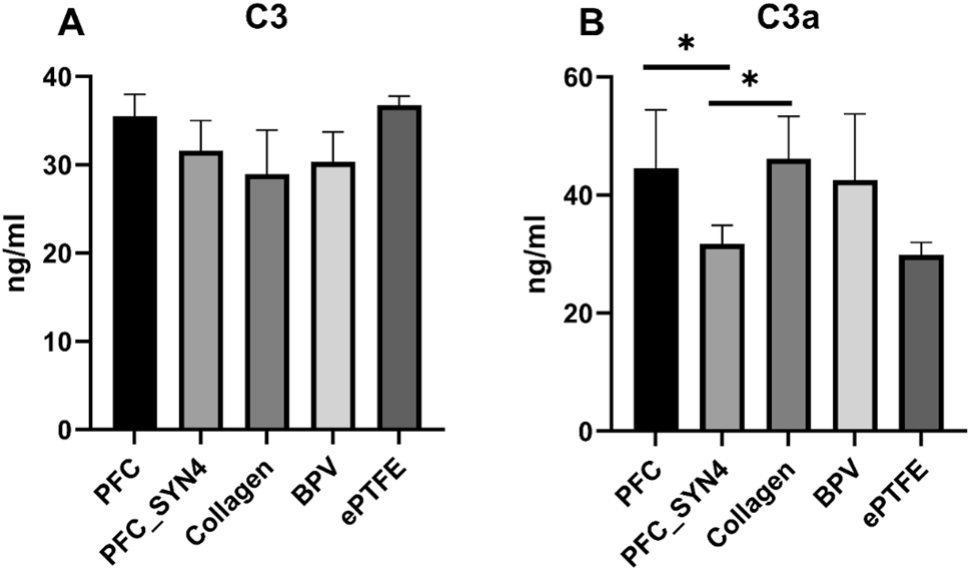
Complement activation on the materials. **A** shows C3 levels and **B** shows C3a levels. BPV and ePTFE were used as controls for the above experiments. (* indicated *P* < 0.05)

Overstimulation of the complement system may result in a variety of noninfectious disorders such as thrombotic, microangiopathic, and cardiovascular graft rejection events. In response to the recognition of a biomaterial surface, a series of complement factors are activated in an enzyme cascade which leads to the generation of a C3 convertase. The C3 convertase cleaves C3 into a small fragment C3a that promotes inflammation and a large fragment C3b that acts as an opsonin. Thus, an increase in C3a amount indicates the activation of the complement system. Complement activation analysis on the materials is shown in Fig. 4A and B. The amount of C3a was significantly lower (*P* < 0.05) in PFC_SYN4, compared to PFC, collagen, and BPV which indicated a lower level of complement activation (Fig. 4B). In this study, PFC-SYN4 had C3a levels comparable to ePTFE and 45% lower compared to collagen and 40% lower compared to BPV.

### Whole Blood Clotting Time

**Fig. 5:**
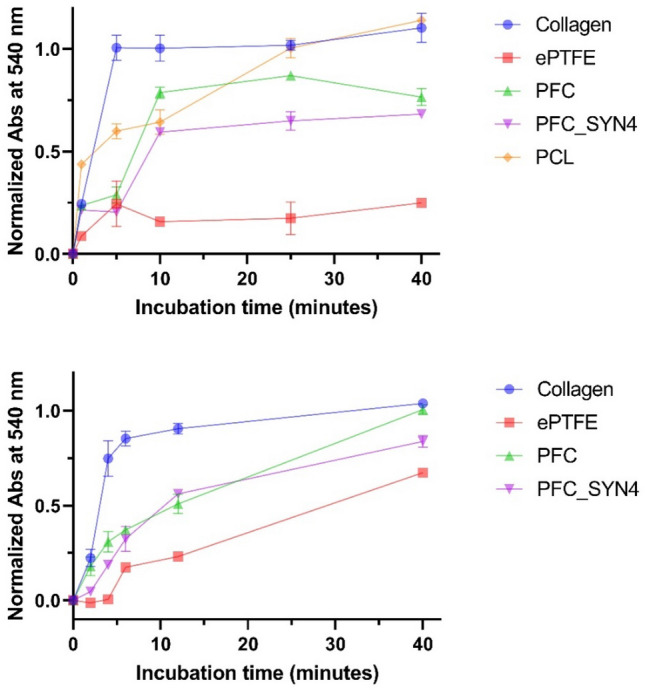
Two experiments of whole blood clotting analysis on PFC, PFC_SYN4, collagen, bovine pericardium valves, and ePTFE. The upper panel had PCL as an extra control group for comparison

The kinetics of whole blood clotting was evaluated to determine the thrombogenicity of syndecan-4 functionalized PFC. Free red blood cells were lysed with distilled water at different time points to assess the extent of clotting. For the upper panel in Fig. 5, ddH_2_O was added to each sample at 1, 5, 10, 25, and 40 min. For the lower panel in Fig. 5, ddH_2_O was added to each sample at 2, 4, 6, 12, and 40 min. Data from the upper and lower panels indicate that blood clotted instantly on collagen, while ePTFE showed a delayed blood clotting time. The clotting process occurred almost instantly for collagen, and at around 5 min of incubation time, the clotting extent was over 90%. ePTFE was the least thrombogenic with the longest incubation time required to reach 50% clotting. As shown in the upper panel, PFC, PFC_SYN4, and ePTFE reached less than 25% clotting while collagen reached 90% clotting after interacting with whole blood for 5 min. PCL, which is commonly used in vascular tissue engineering, showed a thrombogenic tendency. Blood clotted upon contact with PCL within 2 min. Then the clot gradually grew and reached 100% clotting at 25 min, which was similar to collagen.

Whole blood clotting time analysis showed that PFC_SYN4 was less thrombogenic compared with PFC, PCL, and collagen. Additionally, at early time points, from 1 to 10 min as shown in the upper panel and from 2 to 6 min as shown in the lower panel, PFC_SYN4 had a delayed clotting profile compared with PFC (*P* < 0.05). The initial clotting profile is important since it is closely related to the performance of the material after implantation. The results from the two experiments collectively showed that syndecan-4 functionalization reduced the thrombogenicity of PFC. The results suggested the binding of antithrombogenic molecules through the interaction with syndecan-4 covalently linked to the material contribute to the improved hemocompatibility of PFC_SYN4.

## Discussion

There have been ongoing advancements in developing hemocompatible biomaterials ever since blood-contacting medical implants have been used; however, thrombus formation continues to put the patient at risk. Surface hemocompatibility is critical for the short- and long-term patency of any cardiovascular implant, graft, or vascular patch. Of key importance is to engineer biomaterials in a way to minimize inflammation and provide an interaction with blood cells and blood proteins without clot formation.

Heparin has been used to improve the hemocompatibility of vascular biomaterials but is not the ideal solution [21, 22]. Compared with heparin, syndecan-4 used in the present study has a molecular domain and structure similar to syndecan-4 present in native endothelial glycocalyx [25]. The endothelial cell glycocalyx is a viscoelastic layer that is located at the surface of the endothelium [42]. The glycocalyx is in direct contact with the blood and mainly consists of proteoglycans and glycoproteins [43]. This hydrophilic layer serves as a physical barrier between endothelial cells and blood cells and maintains hemostasis. A variety of anticoagulation molecules (such as thrombomodulin, heparin cofactors, and antithrombin) have been shown to bind to specific domains in the glycocalyx [19].

The results of the current study demonstrate that functionalization of a biomaterial, PFC, with syndecan-4 generates a hemocompatible and non-thrombogenic surface that may function, as the glycocalyx of the natural endothelium.

Previous studies in our laboratory have shown PFC to be an elastomeric composite material with compositional, structural, and mechanical similarities to coronary arteries [18]. PFC fabricated as small-diameter grafts had viscoelastic properties and performed mechanically under physiological flow and pressure [34]. In preliminary studies, it was demonstrated that platelets adhered to PFC fibers but were not activated compared to collagen fibers [18]. In the present study, functionalization of PFC with syndecan-4 resulted in a further reduction in platelet interaction. The involvement of platelets as part of the extrinsic coagulation pathway is only one part of the blood coagulation pathway. In this study, the hemocompatibility of syndecan-4 functionalized PFC was examined by investigating other pathways of blood coagulation. Factor XII plays a primary role in initiation of the intrinsic pathway and is essential in surface-activated blood coagulation among the other coagulation factors [44]. No activation of extrinsic or the intrinsic blood coagulation pathway was induced by the functionalization of PFC.

In the present investigation, the complement system was evaluated based on potential interaction and regulation by heparan sulfate of syndecan-4 as has been reported for heparin [45]. Complement system is closely linked to coagulation as a key immunomodulating response upon contacting with biomaterials. Complement C3 is essential for activating the complement system and its cleavage product C3a has been widely used for evaluating the influence of biomaterials on complement activation [46]. The results of the study clearly indicate a significant reduction in complement C3a as a result of the presence of syndecan-4 on the biomaterial. PFC_SYN4 had lower levels of complement activation and reduced thrombogenicity compared with PFC as well as bovine pericardium widely used in cardiovascular surgery. PFC_SYN4 also had similar performance in terms of platelet interaction to commercially available ePTFE grafts. Overall, the results of functionalization of PFC with syndecan-4 demonstrate the potential to regulate thrombus formation at several points in blood coagulation and complement activation pathways. These findings suggest that there is a clinical applicability and benefit of using syndecan-4 to functionalize blood-contacting biomaterial implants. While not a part of the current study, it is possible that syndecan-4 functionalization might improve the long-term performance of biomaterial implants in general through the interaction of heparan sulfate with the molecules critical in maintaining hemostasis. For example, we previously reported that PFC_SYN4 facilitated the binding of stromal cell-derived factor-1alpha and promoted the specific binding and differentiation of endothelial colony-forming cells, all processes that are critical in the formation of a non-thrombogenic endothelium [33, 34].

In this study, PFC_SYN4 had a lower activation level of extrinsic coagulation pathway compared to PFC and collagen as indicated by the reduced extent of platelet activation and a lower number of adhered platelets. These results indicate the non-thrombogenic property of PFC was well maintained after syndecan-4 functionalization. While significantly fewer platelets were bound to PFC_SYN4 based on morphometric findings, there was not total inhibition of platelet binding. Thus, the positive effect of any platelet products may be available but at reduced levels. The addition of syndecan-4 did not result in activation of the intrinsic pathway. The intrinsic pathway is triggered by the interaction of plasma protein with a biomaterial surface. This contact activation leads to the conversion of factor XII to factor XIIa, which is the central member of a self-amplifying activation complex that potentiates the intrinsic coagulation pathway [47]. This may be a result due to the presence of PGS and silk fibroin component in the composite material. Potentially during electrospinning process, the use PGS and silk fibroin may have interfered with the binding sites on collagen for platelets and other proteins involved in the coagulation pathway, thus reducing the thrombogenicity of the PFC. More interestingly, PFC_SYN4 was less thrombogenic compared with PFC as measured by whole blood clotting time, which may work through the interaction of HS chains on syndecan-4 with the endogenous anticoagulation molecules in whole blood, such as antithrombin (AT). Previous studies have identified repeating sequences of specific pentasaccharides found HS with the ability to bind AT with high affinity [48, 49]. If this is the case, the surface density of syndecan-4 might be tuned and optimized to adjust the non-thrombogenicity of the material.

The surface property of biomaterial is closely related to the cell–material interaction and protein–material interaction upon contact with blood. Promoting the hemocompatibility of biomaterials is crucial in clinical settings for many reasons. On the one hand, engineering biomaterial surfaces to improve hemocompatibility may reduce the use of anticoagulation drugs and adverse effects for patients. After surgical intervention, antiplatelet and anticoagulation therapies are usually used to prevent blood clotting on the implants and systemic thromboembolic events. One or a combination of drugs is typically administered to the patients, such as triple therapy (warfarin, aspirin, and clopidogrel), which may prevent acute thrombosis and restenosis [50]. Additionally, to prevent coagulation, the patients may require a daily aspirin for the rest of their lives [51]. These therapies cause undesirable side effects and risks to patients, including acute severe bruising and hemorrhage [52]. On the other hand, generating a hemocompatible biomaterial surface may also reduce the risk of microthrombi accumulations in the circulatory system. The use of syndecan-4 to functionalize biomaterials may enable the development of a variety of blood-contacting cardiovascular implants and medical devices.

## Conclusion

The hemocompatibility of PFC and PFC_SYN4 was evaluated and compared with ePTFE, electrospun collagen, and BPV. Platelets adhered to but were not activated on PFC and PFC_SYN4 materials compared to collagen. The number of adhered platelets on PFC_SYN4 was similar to ePTFE and significantly less compared to PFC, collagen, and BPV. PFC_SYN4 had reduced thrombogenicity compared to PFC and collagen as evaluated by whole blood clotting times. Functionalization of PFC with syndecan-4 resulted in a biomaterial with significantly lower potential to activate complement compared with PFC, collagen, and BPV. These findings suggest PFC_SYN4 is a hemocompatible biomaterial to be used for fabricating native vascular tissue alternatives and improving postoperative graft patency. Ultimately, the use of syndecan-4 to functionalization of other types of biomaterials may be advantageous where regulation with blood and blood components is necessary.

## References

[1] Virani SS, Alonso A, Benjamin EJ, Bittencourt MS, Callaway CW, Carson AP, Chamberlain AM, Chang AR, Cheng S, Delling FN (2020). Heart disease and stroke statistics—2020 update: a report from the American Heart Association. Circulation.

[2] Benjamin EJ, Muntner P, Alonso A, Bittencourt MS, Callaway CW, Carson AP, Chamberlain AM, Chang AR, Cheng S, Das SR (2019). Heart disease and stroke statistics—2019 update a report from the American Heart Association. Circulation.

[3] Fryar CD, Chen T-C, Li X (2012). Prevalence of Uncontrolled Risk Factors for Cardiovascular Disease: United States, 1999–2010.

[4] Harskamp RE, Lopes RD, Baisden CE, De Winter RJ, Alexander JH (2013). Saphenous vein graft failure after coronary artery bypass surgery: pathophysiology, management, and future directions. Ann. Surg..

[5] Suma H (1999). Arterial grafts in coronary bypass surgery. Ann. Thorac. Cardiovasc. Surg..

[6] Stewart SF, Lyman DJ (1992). Effects of a vascular graft/natural artery compliance mismatch on pulsatile flow. J. Biomech..

[7] Berger A, MacCarthy PA, Siebert U, Carlier S, Wijns W, Heyndrickx G, Bartunek J, Vanermen H, De Bruyne B (2004). Long-term patency of internal mammary artery bypass grafts: relationship with preoperative severity of the native coronary artery stenosis. Circulation.

[8] Ivert T, Huttunen K, Landou C, Björk VO (1988). Angiographic studies of internal mammary artery grafts 11 years after coronary artery bypass grafting. J. Thorac. Cardiovasc. Surg..

[9] Wang X, Lin P, Yao Q, Chen C (2007). Development of small-diameter vascular grafts. World J. Surg..

[10] Lawton JS, Tamis-Holland JE, Bangalore S, Bates ER, Beckie TM, Bischoff JM, Bittl JA, Cohen MG, DiMaio JM, Don CW (2021). ACC/AHA/SCAI guideline for coronary artery revascularization: a report of the American College of Cardiology/American Heart Association Joint Committee on Clinical Practice guidelines. Circulation.

[11] Saito J, Kaneko M, Ishikawa Y, Yokoyama U (2021). Challenges and possibilities of cell-based tissue-engineered vascular grafts. Cyborg Bionic Syst..

[12] Gilbert TW, Sellaro TL, Badylak SF (2006). Decellularization of tissues and organs. Biomaterials.

[13] Motlagh D, Yang J, Lui KY, Webb AR, Ameer GA (2006). Hemocompatibility evaluation of poly(glycerol-sebacate) in vitro for vascular tissue engineering. Biomaterials.

[14] Williamson MR, Black R, Kielty C (2006). PCL–PU composite vascular scaffold production for vascular tissue engineering: attachment, proliferation and bioactivity of human vascular endothelial cells. Biomaterials.

[15] Hu J, Sun X, Ma H, Xie C, Chen YE, Ma PX (2010). Porous nanofibrous PLLA scaffolds for vascular tissue engineering. Biomaterials.

[16] van Uden S, Catto V, Perotto G, Athanassiou A, Redaelli AC, Greco FG, Riboldi SA (2019). Electrospun fibroin/polyurethane hybrid meshes: manufacturing, characterization, and potentialities as substrates for haemodialysis arteriovenous grafts. J. Biomed. Mater. Res. B.

[17] Riboldi SA, Tozzi M, Bagardi M, Ravasio G, Cigalino G, Crippa L, Piccolo S, Nahal A, Spandri M, Catto V (2020). A novel hybrid silk fibroin/polyurethane arteriovenous graft for hemodialysis: proof-of-concept animal study in an ovine model. Adv. Healthc. Mater..

[18] Wang R, Levi-Polyanchenko N, Morykwas M, Argenta L, Wagner WD (2015). Novel nanofiber-based material for endovascular scaffolds. J. Biomed. Mater. Res. A.

[19] Ippel BD, Dankers PY (2018). Introduction of nature’s complexity in engineered blood-compatible biomaterials. Adv. Healthc. Mater..

[20] Maitz MF, Martins MCL, Grabow N, Matschegewski C, Huang N, Chaikof EL, Barbosa MA, Werner C, Sperling C (2019). The blood compatibility challenge. Part 4: surface modification for hemocompatible materials: passive and active approaches to guide blood–material interactions. Acta Biomater..

[21] Biran R, Pond D (2017). Heparin coatings for improving blood compatibility of medical devices. Adv. Drug Deliv. Rev..

[22] Patel H (2021). Blood biocompatibility enhancement of biomaterials by heparin immobilization: a review. Blood Coagul. Fibrinolysis.

[23] Capila I, Linhardt RJ (2002). Heparin–protein interactions. Angew. Chem. Int. Ed..

[24] Liang Y, Kiick KL (2014). Heparin-functionalized polymeric biomaterials in tissue engineering and drug delivery applications. Acta Biomater..

[25] Bombeli T, Mueller M, Haeberli A (1997). Anticoagulant properties of the vascular endothelium. Thromb. Haemost..

[26] Xu D, Esko JD (2014). Demystifying heparan sulfate–protein interactions. Annu. Rev. Biochem..

[27] Rabenstein DL (2002). Heparin and heparan sulfate: structure and function. Nat. Prod. Rep..

[28] Kaneider NC, Egger P, Dunzendorfer S, Wiedermann CJ (2001). Syndecan-4 as antithrombin receptor of human neutrophils. Biochem. Biophys. Res. Commun..

[29] Kaneider NC, Reinisch CM, Dunzendorfer S, Römisch J, Wiederman CJ (2002). Syndecan-4 mediates antithrombin-induced chemotaxis of human peripheral blood lymphocytes and monocytes. J. Cell Sci..

[30] Kaneider NC, Förster E, Mosheimer B, Sturn DH, Wiedermann CJ (2003). Syndecan-4-dependent signaling in the inhibition of endotoxin-induced endothelial adherence of neutrophils by antithrombin. Thromb. Haemost..

[31] Tkachenko E, Rhodes JM, Simons M (2005). Syndecans: new kids on the signaling block. Circ. Res..

[32] Billings PC, Pacifici M (2015). Interactions of signaling proteins, growth factors and other proteins with heparan sulfate: mechanisms and mysteries. Connect. Tissue Res..

[33] Warner H, Wu Y, Wagner WD (2021). Syndecan-4 functionalization of tissue regeneration scaffolds improves interaction with endothelial progenitor cells. Regen. Biomater..

[34] Wu, Y., S. K. Yazdani, J. E. M. Bolander, and W. D. Wagner. Syndecan-4 and stromal cell-derived factor-1 alpha functionalized endovascular scaffold facilitates adhesion, spreading and differentiation of endothelial colony forming cells and functions under flow and shear stress conditions. *J. Biomed. Mater. Res. B.* 111(3):538–550, 2023. 10.1002/jbm.b.35170 PMC10092721 36208170

[35] Elfenbein A, Simons M (2013). Syndecan-4 signaling at a glance. J. Cell Sci..

[36] Simons M, Horowitz A (2001). Syndecan-4-mediated signalling. Cell. Signal..

[37] Sotiri I, Robichaud M, Lee D, Braune S, Gorbet M, Ratner BD, Brash JL, Latour RA, Reviakine I (2019). BloodSurf 2017: news from the blood-biomaterial frontier. Acta Biomater..

[38] Wang Y, Ameer GA, Sheppard BJ, Langer R (2002). A tough biodegradable elastomer. Nat. Biotechnol..

[39] Rockwood DN, Preda RC, Yücel T, Wang X, Lovett ML, Kaplan DL (2011). Materials fabrication from *Bombyx mori* silk fibroin. Nat. Protoc..

[40] Warner HJ (2018). Functionalization of a Novel Elastomeric Biomaterial for Vascular Tissue Engineering.

[41] Tamada Y, Kulik EA, Ikada Y (1995). Simple method for platelet counting. Biomaterials.

[42] Cruz-Chu ER, Malafeev A, Pajarskas T, Pivkin IV, Koumoutsakos P (2014). Structure and response to flow of the glycocalyx layer. Biophys. J..

[43] Pries AR, Secomb TW, Gaehtgens P (2000). The endothelial surface layer. Pflüg. Arch..

[44] Weber M, Steinle H, Golombek S, Hann L, Schlensak C, Wendel HP, Avci-Adali M (2018). Blood-contacting biomaterials: in vitro evaluation of the hemocompatibility. Front. Bioeng. Biotechnol..

[45] Merle NS, Church SE, Fremeaux-Bacchi V, Roumenina LT (2015). Complement system part I–molecular mechanisms of activation and regulation. Front. Immunol..

[46] Strohbach A, Busch R (2021). Predicting the in vivo performance of cardiovascular biomaterials: current approaches in vitro evaluation of blood–biomaterial interactions. Int. J. Mol. Sci..

[47] Zhuo R, Siedlecki CA, Vogler EA (2006). Autoactivation of blood factor XII at hydrophilic and hydrophobic surfaces. Biomaterials.

[48] Pejler G, Bäckström G, Lindahl U, Paulsson M, Dziadek M, Fujiwara S, Timpl R (1987). Structure and affinity for antithrombin of heparan sulfate chains derived from basement membrane proteoglycans. J. Biol. Chem>.

[49] Opal SM, Kessler CM, Roemisch J, Knaub S (2002). Antithrombin, heparin, and heparan sulfate. Crit. Care Med..

[50] Sourgounis A, Lipiecki J, Lo TS, Hamon M (2009). Coronary stents and chronic anticoagulation. Circulation.

[51] Goldman S, Copeland J, Moritz T, Henderson W, Zadina K, Ovitt T, Doherty J, Read R, Chesler E, Sako Y (1988). Improvement in early saphenous vein graft patency after coronary artery bypass surgery with antiplatelet therapy: results of a Veterans Administration Cooperative Study. Circulation.

[52] Harter K, Levine M, Henderson SO (2015). Anticoagulation drug therapy: a review. West. J. Emerg. Med..

